# Surface Plasmon Resonance Sensing of Biorecognition Interactions within the Tumor Suppressor p53 Network

**DOI:** 10.3390/s17112680

**Published:** 2017-11-20

**Authors:** Ilaria Moscetti, Salvatore Cannistraro, Anna Rita Bizzarri

**Affiliations:** Biophysics & Nanoscience Centre, DEB, Università della Tuscia, Largo dell’Università, 01100 Viterbo, Italy; i.moscetti@unitus.it (I.M.); bizzarri@unitus.it (A.R.B.)

**Keywords:** Surface Plasmon Resonance (SPR), protein-protein interaction, p53 network, p63, p73, p53 mutants, Azurin, p28, Atomic Force Spectroscopy (AFS)

## Abstract

Surface Plasmon Resonance (SPR) is a powerful technique to study the kinetics of biomolecules undergoing biorecognition processes, particularly suited for protein-protein interactions of biomedical interest. The potentiality of SPR was exploited to sense the interactions occurring within the network of the tumor suppressor p53, which is crucial for maintaining genome integrity and whose function is inactivated, mainly by down regulation or by mutation, in the majority of human tumors. This study includes p53 down-regulators, p53 mutants and also the p53 family members, p63 and p73, which could vicariate p53 protective function. Furthermore, the application of SPR was extended to sense the interaction of p53 with anti-cancer drugs, which might restore p53 function. An extended review of previous published work and unpublished kinetic data is provided, dealing with the interaction between the p53 family members, or their mutants and two anticancer molecules, Azurin and its cell-penetrating peptide, p28. All the kinetic results are discussed in connection with those obtained by a complementary approach operating at the single molecule level, namely Atomic Force Spectroscopy and the related literature data. The overview of the SPR kinetic results may significantly contribute to a deeper understanding of the interactions within p53 network, also in the perspective of designing suitable anticancer drugs.

## 1. Introduction

Since the early 1990s, Surface Plasmon Resonance (SPR) has proven to be one of the most powerful biosensing technique to investigate the recognition processes between biological partners forming functional complexes [1]. The peculiar ability of SPR to determine the kinetic parameters upon physical binding between biomolecules, in real time and without labelling, has made this technique particularly suited for studying interactions in the biomedical field [2]. Interestingly, the kinetic results of protein-protein interactions could provide useful insights into the molecular mechanism at the basis of their biological function. Indeed, SPR has mainly been used to study binding kinetics between antibody-antigen, ligand-receptor and enzyme-substrate, whose interactions are responsible for biomolecular recognition and signaling [3]. In addition, SPR has demonstrated to be a remarkable tool for drug discovery and for diagnostics purposes [4,5]. On the other hand, SPR is able to investigate multiple interactions, such as ternary complexes [6], being useful especially for the study of complex networks, in which many proteins could interact in a combined, synergistic or competitive way. Such a feature is of particular interest for pharmaceutical research looking for drugs antagonizing a specific biological interaction. Indeed, SPR has been exploited to identify the target residues of protein-drug interaction, studying protein domains and/or the effect of mutations and even to measure the drug potency by determining the half-maximal inhibitory concentration [7]. In this connection, the study of protein-protein and protein-drug interactions are at the cutting edge of cancer research. The core of this research is represented by the tumor suppressor p53, which is a powerful transcription factor finely tuned by a complex regulatory network, with a pivotal role in prevention of cancer development and in maintaining genome integrity. In almost all human cancers, p53 function is inactivated mainly by mutations and/or down-regulation; the latter being driven essentially by ubiquitin ligases such as Mouse double minute 2 (MDM2) and Constitutive Photomorphogenic Protein 1 (COP1) [8]. In this context, SPR potentialities to sense interactions within the p53 network has been exploited by many authors, with a particular attention to its down-regulators and also to p53 family members, p63 and p73, which could vicariate p53 function [9,10,11,12,13,14,15,16,17,18,19,20,21,22,23,24,25]. Interestingly, SPR has been also used to search for drugs aimed at protecting the p53 oncosuppressive function, to characterize their binding kinetics and to gain knowledge on their anticancer mechanism [6,26,27,28,29,30,31,32,33,34,35,36,37]. 

Here, we discuss our previous published SPR work dealing with selected protein-protein interactions of p53 or its family members, p63 and p73, with p53 mutants relevant in cancer or with p53 down-regulators. Furthermore, some unpublished results on the binding kinetics of two well-known anticancer molecules, Azurin and its cell-penetrating peptide, p28, with the p53 family members, p53 mutants or some of their domains are provided. At first, the experimental design, including immobilization procedures, kinetic strategies, SPR data and their analysis, is described. The corresponding results are both compared to those obtained by using an emerging biosensing nanotechnological approach, namely Atomic Force Spectroscopy (AFS), being able to measure the unbinding interaction force between biomolecular partners at the level of single molecular complex [38,39,40] and discussed in connection with the related literature data. 

## 2. SPR Principles, Methods and Analysis

### 2.1. SPR Principles 

SPR-based instruments use an optical method to measure a change in the refractive index of the medium in close vicinity (within ~300 nm) of a metal surface. In order to detect an interaction between two molecules, one molecule, the ligand, is immobilized onto the sensor surface, while its binding partner, the analyte, is injected in a buffer solution. As the analyte binds to the ligand, the accumulation of molecules on the surface results in an increase of the refractive index measured by the SPR instrument as a shift in the SPR angle (Resonance angle, °) or quantified in Resonance Units (RU) in Biacore systems (Biacore AB, GE Healthcare, Little Chalfont, UK) (with 1 RU being equivalent to a shift of 10^−4^ degrees) [41]. This change in the refractive index is measured in real time and plotted as response versus time, obtaining the sensorgram. Commercial instruments are equipped with two main liquid handling systems: cuvettes (e.g., SPR Autolab Esprit, Eco Chemie, Utrecht, The Netherlands) or flow cells (e.g., Biacore systems). The former system, which offers the possibility to incubate the analyte over the ligand for long time, can be useful to study slow interactions or even ternary complexes. The main drawback of the cuvette systems is that the open configuration can result in less controlled sample conditions, allowing uncontrolled evaporation of the sample and increase in salt concentration, than the flow cell systems. On the other hand, these systems are characterized by carefully defined experimental conditions, then, providing more accurate kinetic parameters. However, in the latter case the contact between analyte and ligand is limited by both the injection volume and the flow rate [42].

### 2.2. Immobilization Strategies

The immobilization of a protein ligand over a sensor surface is based on two main strategies; one involving a direct binding of the ligand to the surface by covalent coupling, the other using an indirect immobilization through the high affinity capture of the ligand by a covalently coupled molecule. There are three main types of covalent coupling chemistry, using the amine group of lysines, the thiol group of cysteines or the aldehyde group of carbohydrates, to covalently bind proteins to sensor chip surfaces exposing free carboxymethyl groups, such as CM-series chips (GE Healthcare). The most used chemistry is the amine coupling which consists of the activation of the carboxymethyl groups by *N*-ethyl-*N*-(3-diethylaminopropyl) carbodiimide (EDC) and *N*-hydroxyl-succinimide (NHS) to give reactive succinimide esters, which spontaneously react with protein amines to form covalent links. An alternative strategy, using bare gold SPR sensor disks, involves the gold surface functionalization with cysteamine and glutaraldehyde, prior to the covalent binding of proteins through amine coupling. These direct immobilization approaches don’t need any ligand modification but cause the immobilization of the ligand in different orientations. Some of these orientations may have a negative effect by decreasing or even abrogating the ligand ability to bind to the analyte. In addition, an efficient ligand regeneration could be difficult to be achieved. On the other hand, in the indirect immobilization strategy, the ligand needs to have a suitable binding site or a tag allowing it to be captured with a high specificity and to be effectively dissociated by regeneration procedures. The most used strategies involve antibody capture of tags, such as GST (e.g., GST Capture kit, GE Healthcare), usually linked to the *N*-terminus of recombinant proteins. This strategy has important advantages since proteins are rarely inactivated by indirect coupling and all the molecules are immobilized in a known and well determined orientation on the surface. In addition, by using appropriate buffers, the captured ligand-analyte bond can be selectively dissociated, thereby enabling the surface to be re-used. In any case, a control surface should be generated, being as similar as possible to the ligand surface, to measure non-specific binding and to record the background response. 

### 2.3. Kinetic Experiments and Data Analysis

The classical method of measuring binding constants by SPR involves testing several analyte concentrations over the same ligand surface and regenerating the surface between each analyte injection cycle; this strategy is defined as Multi-Cycle Kinetics (MCK). It can also be adapted to study the effect of inhibitors over an interaction, while its main drawback is the difficulty to regenerate the ligand without affecting the ability to bind the analyte in the successive injection cycle. An alternative approach, called Single-Cycle Kinetics (SCK), consists in sequential injections of increasing concentrations of the analyte over a functionalized sensor chip surface, without regeneration steps between each sample injection [43]. This method can be more efficient than the MCK and allows to fully characterize analyte binding to ligand surfaces that are difficult to be regenerated. 

To extract the kinetic parameters, the SPR data of protein-protein interaction are usually analyzed in the framework of the Langmuir 1:1 binding model, which assumes a simple reversible bimolecular reaction between the ligand and the analyte [44,45]. The model is modified to take into account for the mass transport effect [46] by assuming that the analyte is driven towards the sensor chip surface (*A_surface_*) or back again to the bulk solution (*A_bulk_*) with the same mass transfer rate constant (*k_t_*). When the analyte reaches the sensor chip surface, it binds to the ligand resulting in the formation of the ligand-analyte complex (*LA*), characterized by the association rate constants (*k_on_*) and the dissociation rate constants (*k_off_*):

(1) $$A_{bulk}\;\overset{K_{t}}{\underset{K_{t}}{⇄}}\;A_{surface}\;+\;L\;\overset{K_{on}}{\underset{K_{on}}{⇄}}\;LA$$

Accordingly, the variation of *A_surface_*, *L* and *LA* concentrations with time can be described by the following set of differential equations [47]:

(2) $$\frac{d\left[A_{surface}\right]\;}{dt}\;=\;k_{t}\;\left(\left[A_{bulk}\right]-\left[A_{surface}\right]\right)\;-\;\left(k_{on}\left[L\right]\left[A_{surface}\right]-k_{off}\left[LA\right]\right)\;\frac{d\left[L\right]}{dt}=-(k_{on}\left[L\right]\left[A_{surface}\right]-k_{off}\left[LA\right])\;\frac{d\left[LA\right]}{dt}(k_{on}\left[L\right]\left[A_{surface}\right]-k_{off}\left[LA\right])$$

where [*A_bulk_*] is the analyte concentration in bulk solution, [*A_surface_*] is the analyte concentration at the sensor chip surface, [*L*] is the ligand concentration and [*LA*] is the ligand-analyte complex concentration. By fitting the sensorgram according to a non-linear least square analysis and numerical integration of Equations (2) through the BiaEvaluation software or other program (e.g., CLAMP software [48]), the kinetic parameters *k_on_* and *k_off_* can be determined, then the equilibrium dissociation constant, *K_D_*, (*K_D_* = *k_off_/k_on_*) can be calculated.

On the other hand, the *K_D_* can be also derived by plotting the response at equilibrium (*R_eq_*) versus the analyte concentration [*Analyte*] and then fitted as a Langmuir isotherm [44,45]:

(3) $$R_{eq}=\frac{\;\left[Analyte\right]R_{max}}{K_{D}+\left[Analyte\right]}+RI$$

where *R_max_* is the analyte binding capacity and *RI* is the bulk refractive index contribution of the sample, which is assumed to be the same for all the injections and used as the Response-axis offset. 

## 3. Kinetics of Protein—Protein Interactions within the p53 Network by SPR

### 3.1. The Interaction between p53 and its Main Down-Regulator, MDM2

**Figure 1:**
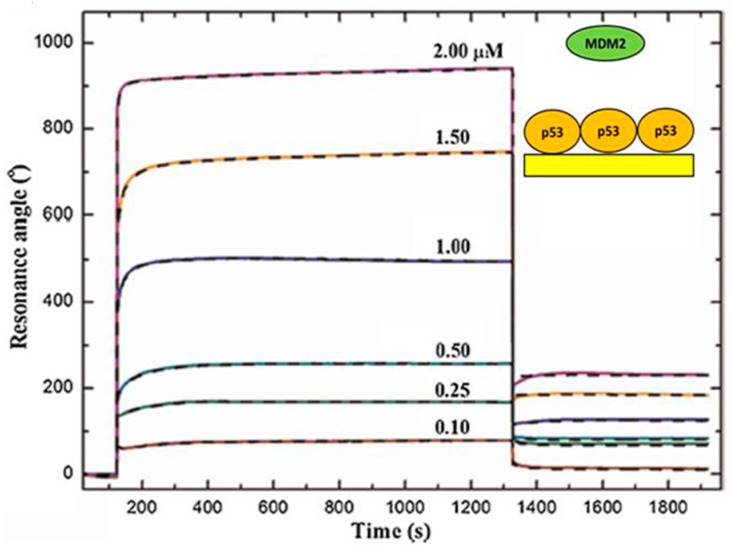
Sensorgrams (solid curves) of the Multi-Cycle Kinetics performed by injecting over the p53-functionalized substrate increasing concentrations of MDM2. Dashed black curves: the best fits of experimental data with a 1:1 binding model (CLAMP software). Insets: schematic representation of the interaction geometry. Adapted from [22].

The interaction of the tumor suppressor p53 with MDM2, the major E3 ubiquitin ligase driving p53 to proteasome for degradation, is of outstanding interest and this interaction is considered as one of the main targets for anticancer drug design aimed at impairing p53 down-regulation [49]. To characterize the kinetic details of such an important interaction, the formation of the MDM2-p53 complex was studied by using SPR [22]. The Autolab Esprit instrument was used to perform a MCK with MDM2 analyte over a sensor disk functionalized with cysteamine and glutaraldehyde to covalently bind the p53 ligand through its exposed lysines. Figure 1 shows SPR sensorgrams obtained from the injection of MDM2 protein, at six different concentrations (ranging from 0.1 to 2 µM), on the sensor disks covered by p53. The SPR signal, as a function of time, provides the binding kinetic characterization of the complex. Upon MDM2 injection, the observed time dependent signal increases up to a plateau as due to the MDM2-p53 association; after removal of the MDM2 solution and subsequent buffer injection, the decreasing profile reflects the kinetics of the MDM2-p53 dissociation. A *k_off_* of about 1 s^−1^, with a corresponding lifetime (*τ = 1/k_off_)* in the order of 1 s and a *K_D_* of about 10^−7^ M were obtained, according to the 1:1 binding model (CLAMP software) (Figure 1A, Table 1). Interestingly, these kinetic results were further confirmed by complementary experiments performed at the single molecule level by using AFS providing a *k_off_* of about 1.5 s^−1^ [50]. Moreover, similar *K_D_* values were obtained by other authors for the interaction between the *N*-terminal domain (NTD) of MDM2 and the full length p53 or the NTD of p53, witnessing that both their NTDs are mainly responsible for the MDM2-p53 interaction [51,52]. Such information on the kinetics of the MDM2-p53 interaction could help to understand the molecular mechanism underlying the inhibitory function exerted by MDM2, also in connection with the kinetics of other competitive or synergistic interactions within the p53 network. 

### 3.2. The Interaction between MDM2 and MDM4

**Figure 2:**
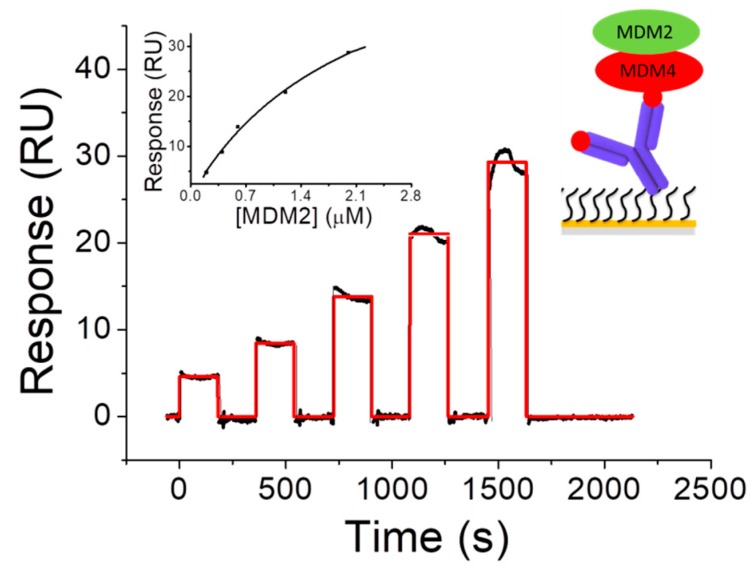
Sensorgram (black curve) of the Single-Cycle Kinetics performed by injecting of five increasing concentrations (in the 0.2–2.0 µM range) of MDM2 over the MDM4-functionalized substrate. Red curve: best fit of the sensorgram with the 1:1 binding model (BiaEvaluation software). Left inset: plot of the response at equilibrium versus the MDM2 concentration. Black curve: fit of the experimental data with the Langmuir isotherm (Equation (3)). Right inset: representation of the interaction scheme. Adapted from [23].

The interaction of MDM2 with its homolog MDM4, forming heterodimers, plays a pivotal role in the p53 network; primarily by controlling p53 abundance through ubiquitin-proteasome pathway and also because of its involvement in the regulation of p53 transcriptional activity and p53-induced apoptosis [53,54,55]. Therefore, the MDM2-MDM4 complex could be a target for promising therapeutic restoration of p53 function [56]. To this aim, a deeper understanding of the molecular mechanisms underlying the heterodimerization is required. In this context, the kinetics of the MDM2-MDM4 interaction was characterized by SPR [23]. An immunocapture strategy was exploited to immobilize N-terminal GST-tagged MDM4 by using the Anti-GST Antibody (right inset of Figure 2). This immobilization strategy allowed to orient MDM4 in a convenient way, by exposing the C-terminal RING domain, which is involved in the interaction with MDM2 [57,58]. The BiacoreX100 system was exploited to perform a SCK in which the MDM2 analyte and the buffer were alternately injected into the flow cell, where the MDM4 ligand was previously immobilized. The sensorgram (Figure 2) shows the SPR response (RU) as a function of time obtained by the successive injection of five increasing concentrations of MDM2. During the first injection, the signal increases reaching a steady state before the end of the injection. Subsequently, the MDM2 rapidly and completely dissociates as the signal strength decreases, down to zero. The same trend is also observed for the successive injections of increasing concentration of MDM2. To extract information on the affinity between MDM2 and MDM4, the SPR data were analyzed in the framework of the 1:1 binding model by using the BiaEvaluation software (version 2.1, Biacore AB, GE Healthcare, Little Chalfont, UK) and a koff of about 10^−3 s−1^, a corresponding lifetime in the order of minutes and a KD of about 10^−7^ M, were obtained (Figure 2, Table 1). A slightly higher KD value (about 10^−6^ M) was found by fitting the response at equilibrium versus the MDM2 concentration with the Langmuir isotherm (Equation (3)) (left inset of Figure 2). This KD value was further confirmed at the single molecule level by AFS which, instead, provided a higher koff (about 10^−2^ s^−1^), corresponding to a slightly shorter lifetime [23]. Such a difference between the results from in bulk experiments by SPR and at the single molecule level by AFS, could be attributed to the peculiar features of the two experimental techniques and also to the different immobilization strategies [39]. 

Interestingly, the lifetime of MDM2-MDM4 interaction is much longer than that of the p53-MDM2 complex (Table 1), with this indicating that the heterodimer is available for several cycles of association and dissociation with p53 before the displacement of the MDM2-MDM4 complex occurs. The longer lifetime of the MDM2-MDM4 complex, with respect to that of MDM2-p53, could be consistent with the efficacy of the heterodimer in the p53 down-regulation. These new insights into the kinetics of the MDM2-MDM4 complex may contribute to a better understanding of the ternary complex formed by the MDM2-MDM4 heterodimer and p53 and, more importantly, could be of significant help in designing specific antagonists able to prevent the formation of the MDM2-MDM4 complex, thus protecting p53 oncosuppressive function.

### 3.3. The Interaction of p53 Family Members with the Oncogenic Mutant p53R175H

In case of p53 inactivation, other members of p53 family, namely p63 and p73, which share high structural homology with p53, are able to vicariate the oncosuppressive function of p53 by regulating cell proliferation, differentiation and apoptosis [59,60]. Unfortunately, some mutants of p53, such as p53R175H which is frequently found in many tumors, such as colorectal and breast cancers [61], inhibit the anti-tumor function of both p63 and p73 [62,63,64,65,66]. Accordingly, the elucidation of the binding kinetics of the complexes formed by p53R175H and p53 family members, might contribute to the design of novel anticancer drugs which could antagonize p53R175H and make p63 or p73 available for anti-tumor effects. Furthermore, the oncogenic mutant p53R175H is able to impair the wild type p53 tumor suppressive function even when this is still present [67], although the mechanism underlying such a dominant negative effect is still highly debated [66,68,69,70]. 

On such a ground, the kinetics of p53R175H-p73, p53R175H-p63 and p53R175H-p53 complexes was studied by SPR [24,25]. In all the cases, a SCK was performed by using the BiacoreX100. Increasing concentrations of p53R175H analyte were injected over a substrate functionalized with p73 by amine coupling or with GST-tagged p63 or p53 by immunocapture, used as ligand. Sensorgrams witnessed the formation of specific complexes between p53R175H and all the p53 family members; a fitting of these data with the 1:1 binding model (BiaEvaluation software) provided the kinetic parameters (Table 1). In particular, the p53R175H-p63 complex, characterized by a *K_D_* value in the order of 10^−9^ M and a *k_off_* value of about 10^−5^ s^−1^, is stronger than the complex formed by the same mutant with p73, whose *K_D_* value is in the order of 10^−7^ M and *k_off_* value is of about 10^−3^ s^−1^. Indeed, these differences could be traced back to the presence of a specific aggregating peptide identified in the p63 sequence [68], which might drive the stronger interaction with p53R175H.

Notably, the p53R175H-p53 complex is characterized by high specificity (*k_off_* ≈ 10^−5^ s^−1^) and high affinity (*K_D_* ≈ 10^−9^ M), similarly to what found for the p53R175H-p63 complex (Table 1). Indeed, this good agreement could indicate that the molecular mechanism underlying the formation of both p53R175H-p53 and p53R175H-p63 complexes is similar [66]. All these kinetics results were further confirmed by AFS experiments indicating a strong and specific interaction of the mutant p53R175H with all the p53 family members (*k_off_* ≈ 10^−5^ s^−1^), also at single molecule level [24,25]. The only exception is the *k_off_* value of the p53R175H-p73 complex, found by AFS, which is significantly lower respect to that obtained by SPR but more similar to those of the other p53 family-p53R175H complexes. Interestingly, by comparing the kinetic parameters of the p53R175H-p53 complex with those reported for the p53-p53 homodimer interaction [71], crucial for the oncosuppressive function of p53 in vivo [72], we note a comparable high affinity. However, the dissociation rate of the homodimer is much faster than that of the p53R175H-p53 complex. In this context, the observed p53R175H-p53 interaction could antagonize the homodimer formation, especially when high levels of p53R175H accumulates in cancer cells.

**Figure 3:**
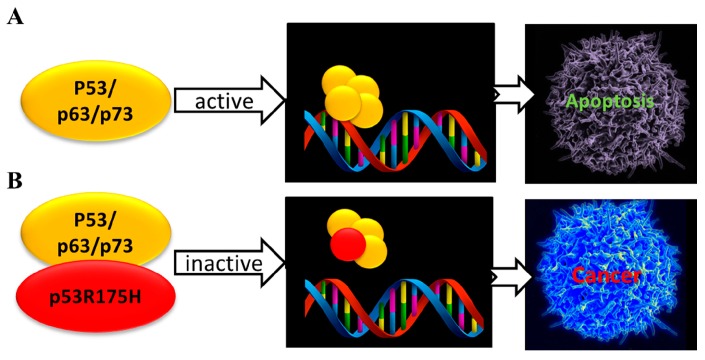
Schematic representation of the p53 family function (**A**) and its inactivation after their binding with the oncogenic mutant p53R175H (**B**). The active p53 family proteins drives transactivation and finally apoptosis of damaged or aberrantly proliferating cells. Binding of p53R175H with p53 family inhibits the pro-apoptotic transactivation with loss of the p53 family tumor suppressive function.

Collectively, the strong interaction of the mutant p53R175H with all the p53 family members could trigger the sequestering of the p53 family members. This may lead to the dominant negative effect shown by this mutant, which finally causes the inhibition of the pro-apoptotic transactivation function and loss of the p53 family protective function; with these effects being connected with oncogenic outcomes (Figure 3).

### 3.4. The Interaction of p53 with the Anticancer Molecule Azurin and the Effect on the p53-MDM2 Binding 

**Figure 4:**
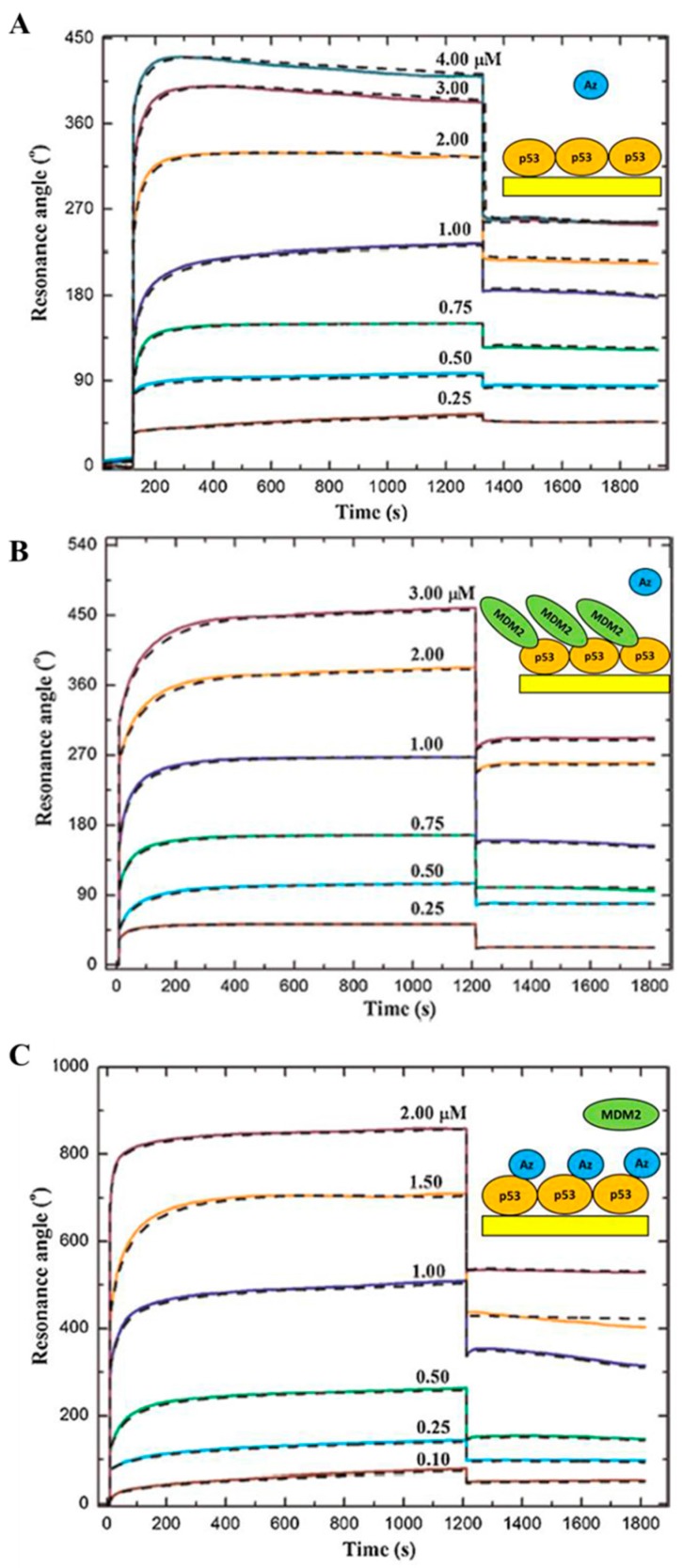
Sensorgrams (solid curves) of the Multi-Cycle Kinetics performed by injecting over the p53-functionalized substrate increasing concentrations of: (**A**) Azurin, (**B**) Azurin, after the substrate saturation with MDM2, (**C**) MDM2, after the substrate saturation with Azurin. Dashed black curves: the best fits of experimental data with a 1:1 binding model (CLAMP software). Insets: schematic representation of the interaction geometry. Adapted from [22].

The bacterial blue copper protein Azurin plays a prominent anticancer role by entering cancer cells and inducing their apoptotic death [73]. Interestingly, it has been demonstrated that this pro-apoptotic action of Azurin is concomitant with the formation of a complex with p53, thereby leading to both its stabilization and intracellular level increase [74]. Taking in mind that further biophysical studies may assist the design of novel Azurin-based cancer treatments, the kinetic parameters of Azurin-p53 interaction were investigated by SPR. In particular, the same strategy described above for the MDM2-p53 complex was used; the p53 ligand was covalently immobilized by amine coupling over a functionalized sensor disk, then a MCK with the Azurin as analyte was performed by using the SPR Autolab Esprit. The SPR sensorgrams, shown in Figure 4A, were obtained by injecting Azurin at seven different concentrations, ranging from 0.25 to 4 µM, onto the p53-functionalized substrate. By fitting the sensorgrams with the 1:1 binding model (CLAMP software), a *k_off_* of about 10^−1^ s^−1^ and a *K_D_* of about 10^−6^ M were found (Table 1). These binding parameters are consistent with the formation of a specific Azurin-p53 complex, with the *k_off_* value being in good agreement with that obtained by AFS for the same complex at the single molecule level [75]. In addition, the *N* terminal domain (NTD) of p53 has been reported to maintain a similar ability to bind Azurin [76]. Interestingly, the dissociation constant of the Azurin-p53 complex results to be only threefold lower than that of the MDM2-p53 complex (Table 1). 

Furthermore, SPR analysis of the Azurin-p53-MDM2 ternary interaction, whose occurrence has been proposed by AFS experiments, was performed [50]. Indeed, aiming at extracting the relevant kinetic parameters and at ascertaining if the observed Azurin-p53 specific interaction could interfere with the binding kinetics of the MDM2-p53 complex, the binding kinetics of both Azurin with the MDM2-p53 complex and of MDM2 with the Azurin-p53 complex was investigated. For the first binding configuration, p53-functionalized substrate was treated with an excess of MDM2 solution until the binding capacity of p53 for MDM2 resulted totally reduced. Then, the Azurin solution was injected on the MDM2-p53 complex immobilized on the SPR sensor disk to analyze the interaction kinetics. The sensorgrams, shown in Figure 4B, were obtained by injecting six different concentration of Azurin; the extracted kinetic rate constants and equilibrium dissociation constant are almost equivalent to those obtained for the Azurin-p53 binary complex (Table 1). These results indicate that Azurin and MDM2 do not compete for the same binding site within p53 but they are involved in a ternary interaction (Azurin-p53-MDM2), confirming previous observations at the single molecule level [50]. The same strategy was used to investigate the ability of MDM2 to bind to the Azurin-p53 complex (Figure 4C). 

**Figure 5:**
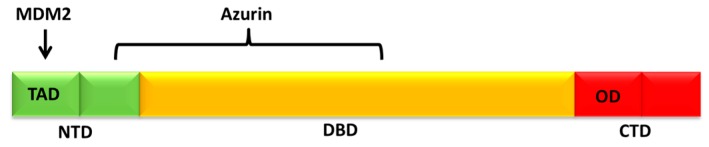
Schematic representation of the full length p53 protein domains: *N*-terminal domain (NTD), containing the transactivation domain (TAD), DNA binding domain (DBD) and C terminal domain (CTD), including the oligomerization domain (OD). The arrow indicates the known interacting domain of p53 with MDM2 and the interacting regions of p53 with Azurin are marked.

By comparing with the MDM2-p53 binary interaction, we found that Azurin affects the interaction of MDM2 with p53 by reducing of more than four times the corresponding association rate; whereas the corresponding *k_off_* value is practically unchanged and, consequently, the resulting *K_D_* value is increased (Table 1). In other words, the SPR results indicate that the specific binding of Azurin to p53 induces a lowering of the association kinetics and binding affinity of the MDM2-p53 complex, without obstructing the MDM2 binding site on p53. In this connection, Azurin can bind to the *N*-terminal domain (NTD) of p53 [73,75,77] but not to the transactivation domain (TAD) of p53 [76], where the interaction with MDM2 occurs [78,79] (Figure 5). In addition, several experimental and computational studies showed a direct contact of Azurin with the DNA binding domain of p53 (DBDp53) [73,80,81]. In any case, Azurin could somehow affect the MDM2-p53 recognition process without hindering the accessibility of MDM2 to its binding pocket on p53 but inducing a weakening of the MDM2-p53 interaction through a non-competitive inhibition mechanism; this could be figured out as a long-range binding regulation. Moreover, the Azurin-induced folding in p53, as evidenced by Circular Dichroism (CD) measurements, suggests that Azurin could be able to stabilize p53 by allosteric inhibition of the functional, regulative interaction between MDM2 and p53 [22]. This non-competitive modulation of the p53 activity may represent an interesting p53-protective strategy to design anticancer drugs to treat tumors in which p53 retains its wild-type structure and function and provides enlightening insights into the mechanism underlying the observed anticancer action of Azurin.

### 3.5. The Interaction of the Anticancer Drug p28 with p53 

**Figure 6:**
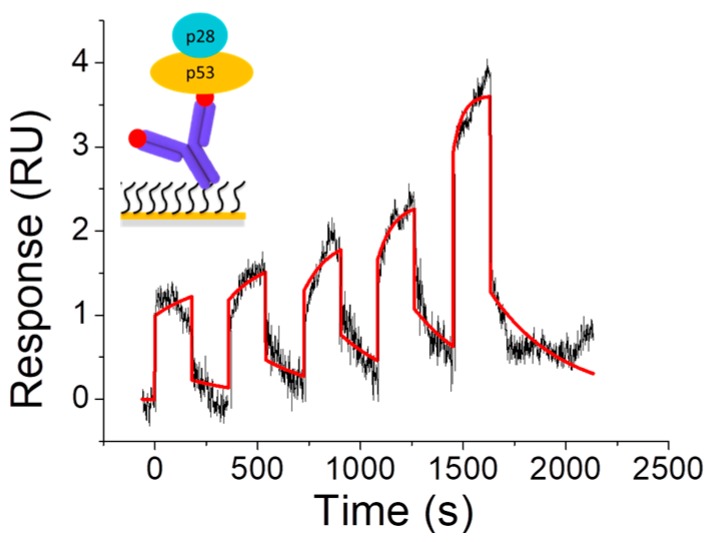
Sensorgram of the Single-Cycle Kinetics performed by injecting five increasing concentrations (5, 10, 20, 40, 80 nM) of p28 over the p53-functionalized substrate (black curve). Red curve: best fit of the sensorgram with the 1:1 binding model (BiaEvaluation software). Inset: representation of the interaction scheme.

p28 is a cell-penetrating peptide derived from Azurin, which causes a post-translational increase of p53 in cancer cells and is a promising drug [82]. Indeed, it passed Phase I clinical trial on adult solid tumor and was found safe for children [83,84]. Interestingly, p28 directly binds p53, without altering p53 overall conformation [85,86,87]. In this context, the molecular mechanism of p28 anticancer activity is a remarkably interesting topic to understand the function and possibly to improve the anticancer properties of such this drug. To this aim SPR experiments were performed to investigate the p28 binding to the full length p53 protein (p53) and to some of its domains, which could be responsible for the interaction with p28 [37]. The GST Capture kit was exploited to immobilize the GST-tagged p53 protein over a CM5 sensor chip surface and a SCK with the p28 analyte was performed by using the Biacore X100 instrument. Figure 6 shows the sensorgram obtained by injecting five increasing concentration of p28 over the p53-functionalized surface by using the method described in detail in [37]. After each injection of p28, the response increases, then, after the buffer flows, it rapidly decreases. Indeed, the response grows after the injection of higher concentrations of p28, indicating the occurrence of a specific interaction between p28 and p53. 

By fitting the sensorgram with the 1:1 binding model (BiaEvaluation software), we found a *k_off_* of about 10^−3^ s^−1^, a corresponding lifetime in the order of minutes and a *K_D_* of about 10^−5^ M (Figure 6, Table 1), indicating the occurrence of a specific and quite strong interaction between p53 and p28. This affinity is similar to that obtained for the p28-p53 complex, at the single molecule level, by AFS (*K_D_* ≈ 10^−5^ M). At variance, slightly lower *k_off_* was found by SPR respect to AFS (*k_off_* ≈ 10^−1^ s ^−1^) [87], similarly to what found for the MDM2-MDM4 complex. By comparing these results with those obtained for the Azurin-p53 complex, we found a few lower affinity and a significantly longer lifetime; with this being an important feature for the anticancer activity of p28 respect to the whole Azurin (Table 1).

Furthermore, since biological studies, as well as computational methods, indicated the DBD as the interacting domain of p53 with p28 [85,87,88], the kinetics of the complex between p28 and the DBDp53 was investigated by SPR [37]. The GST-tagged DBDp53 ligand was immobilized by using the same capture strategy of the whole p53 protein; then, a SCK was performed by injecting increasing concentrations of p28 analyte. The sensorgram, shown in [37], reveals the occurrence of a specific interaction between p28 and DBDp53. By fitting this sensorgram with the 1:1 binding model (BiaEvaluation software), a *k_off_* of about 10^−5^ s^−1^, a corresponding lifetime in the order of hours and a *K_D_* of about 10^−7^ M (Table 1) were determined. These results qualitatively confirm the binding properties detected by AFS, showing quite similar *K_D_* values but a lower *k_off_* respect to AFS (*k_off_* ≈ 10^−2^ s^−1^) [87]. Interestingly, the DBDp53 showed a higher affinity and a longer lifetime with respect to the full length p53 protein (Table 1). 

The same SPR approach was also used to study the interaction of p28 with the C-terminal domain (CTD) of p53. However, in this case, no specific response was found, confirming that this domain of p53 is not involved in p28 interaction, as previously shown by immunoprecipitation assays [85]. The interaction of p28 with the *N*-terminal domain of p53 was not tested since no interaction was found by AFS [87].

Collectively, these kinetic results strengthen and complete previous findings on the p28-p53 complex, confirming that the interaction of p28 is confined to the DBD core domain through formation of a high affinity complex. The occurrence of a strong complex between the DBDp53 and p28 suggests that the p28 anticancer activity may be related to its ability to inhibit the binding of E3 ligase COP1 to the DBD by reducing the proteasome degradation of p53 [85]. In this respect, SPR competitive assays of COP1 over the DBDp53-p28 complex, performed by using a suited strategy, such as that described above for MDM2 over the Azurin-p53 interaction, would provide significant insight into the anticancer function of p28.

### 3.6. The Interaction of Mutants of DBDp53 with p28 

**Figure 7:**
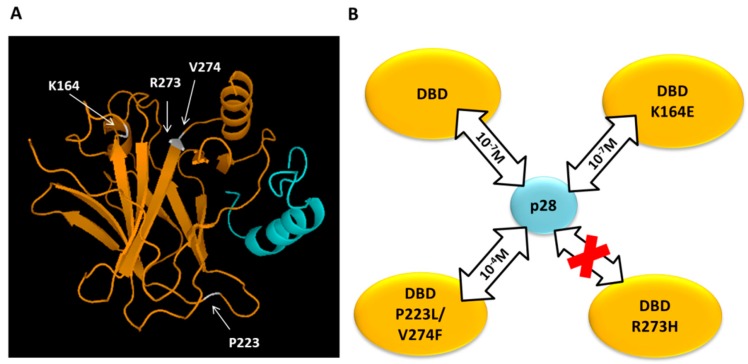
(**A**) DBDp53-28 docking model: in yellow, ribbon diagram of wild type DBDp53, mutated residues are indicated by arrows and highlighted in grey; in cyan, ribbon diagram of p28. Adapted from [85]. (**B**) Schematic representation of the binding abilities of p28 respect to the wild type DBDp53 (DBD) and the three mutant DBDs (K164E, R273H and P223L/V247F); in case of interaction, the *K_D_* value is indicated inside the arrow.

The mutation of p53 occurs in half of human cancers and is mainly located in the DBD [61], which is the binding site of p28, as discussed above. The DBD is not only involved in the control of the p53 down-regulation but it is the domain necessary for the binding to DNA. Since mutations within DBD are often connected with p53 loss of function and subsequent tumor proliferation, it could be interesting to investigate the possibility that p28 could interact with mutated forms of DBD enhancing apoptosis. In this connection, the ability of p28 to bind to naturally occurring DBD mutants was investigated. In particular, the mutants K164E DBDp53, R273H DBDp53 and P223L/V274F DBDp53, whose mutation sites are not overlapping the p28 binding sites, were used for SPR experiments [37] (Figure 7A). The SCK approach (BiacoreX100) was used to study the interaction kinetics between mutants DBDp53 immobilized on the SPR sensor chip surface and p28 in solution by using the same strategy, above described for the p28-DBDp53 wild type. When increasing concentrations of p28 were fluxed over the K164E DBDp53 or P223L/V274F DBDp53 functionalized surface, responses similar to that of the p28-DBDp53 wild type were found. No increase in signal was observed when the p28 was injected over the R273H DBDp53 functionalized surface (data not shown). These kinetic data were analyzed in the framework of the 1:1 binding model (BiaEvaluation software). Accordingly, a *K_D_* value of about 10^−7^ M was determined for the p28-K164E DBDp53 interaction, indicating a strong affinity which is very close to that of the wild type one (Table 1). Indeed, the p28-P223L/V274F DBDp53 interaction, which is characterized by a *K_D_* ≈ 10^−4^ M, showed a lower affinity with respect to the complex between the former mutant and the wild type DBDp53 (Table 1). Instead, no interaction between p28 and the R273H DBDp53 was found (Figure 7B). These kinetic results were further confirmed, at the single molecule level, by AFS and correlated to information on the secondary structure of DBD mutants affecting p28 binding. Indeed, Raman spectroscopy pointed out that the affinity of p28 with DBDp53 mutants is correlated with the β-sheet content [37]. Additionally, these results could provide insights on how p28 plays an anticancer activity in patients with a variety of advanced tumors presenting wild type or mutated p53, as evidenced in initial clinical trials [83,84]. 

### 3.7. The Interaction of p53 Family with Azurin and p28 

The structural similarity between p53 and its homologues p63 and p73, especially concerning their DBDs, suggests that anticancer molecules able to bind to p53 could also bind to either p63 or p73 and potentially enhance their tumor suppressive activity. In this context, the ability of Azurin to bind p63 was investigated by SPR measurements. In particular, a SCK (Biacore X100) was performed by injecting Azurin, in a concentration range of 5–50 µM, over a CM5 sensor chip surface functionalized with the DBD of p63 (DBDp63) by using the amine coupling strategy. The increase of the SPR response, as a function of the Azurin concentration, indicates a specific binding of Azurin with the DBDp63 (data not shown). The kinetic parameters of the Azurin-DBDp63 interaction, analyzed in the framework of the 1:1 binding model (Table 1) and also of the Langmuir isotherm (*K_D_* = (3.9 ± 0.8) × 10^−5^ M) were determined. In particular, the *K_D_* value of the Azurin-DBDp63 complex (≈10^−5^ M) indicates a quite strong biorecognition, being weaker than the Azurin-p53 complex (Table 1). Since the DBDp63 shares high sequence homology with the DBDp53 and is able to bind to Azurin, its ability to bind to p28 was further investigated. A SCK (BiacoreX100) was performed by injecting 5 increasing concentrations of p28 (3–13 µM) over the p63DBD-immobilized surface, prepared as described above. The sensorgram (data not shown) indicated that p28 specifically binds to the DBDp63 according to the 1:1 binding model (BiaEvaluation software) their kinetic parameters were determined (Table 1). The p28-DBDp63 complex is characterized by a quite high affinity (*K_D_* ≈ 10^−6^ M) and a very long lifetime in the order of hours. In addition, by fitting the SPR response with the Langmuir isotherm (Equation (3)) (BiaEvaluation software), a similar *K_D_* value (*K_D_* = (7.2 ± 0.8) × 10^−6^ M) was found. Furthermore, the resulting affinity is also close to that previously obtained at the single molecule level by AFS (*K_D_* ≈ 10^−6^ M), while the lifetime of the p28-DBDp63 appears longer [89]. Such a difference between the results of these two techniques was already shown for the MDM2-MDM4, the p28-p53 and the p28-DBDp53 complexes. By comparing the kinetic results of the complex formed by DBDp63 with p28 or with the Azurin, a few higher affinity and a longer lifetime with the p28 peptide were found (Table 1). On the other hand, the p28-p63DBD complex is less stable than the cognate p28-DBDp53 complex. These kinetics results may have implications on the possible efficacy of such these anticancer molecules over p63 function, opening new insights on their biological function. The longer lifetime of the interaction of p63 with p28 respect to Azurin, also shown for p53, may be an important feature of their anticancer activity. 

To complete the study of p28 abilities to bind to the p53 family members, its interaction with p73 was studied. By using the BiacoreX100 instrument, a SCK was performed by injecting increasing concentrations of p28 analyte (in the 3–12 µM range) over a CM5 sensor chip surface functionalized with the p73 ligand by using the amine coupling chemistry. The sensorgram was fitted with the 1:1 binding model (BiaEvaluation software); the resulting kinetic parameters indicate a high affinity (*K_D_* ≈ 10^−8^ M) and a lifetime in the order of minutes for the p28-p73 complex (Table 1). By comparing these kinetic results with those obtained, at the single molecule level by AFS (*K_D_* ≈ 10^−7^ M) [89], a comparable lifetime and a few higher affinity were found by SPR. Intriguingly, the p28-p73 complex is characterized by higher affinity but a lower stability, than the p28-p53 complex.

Collectively, these novel SPR kinetic results confirm that p28 is able to form stable and high affinity interactions with both p63 and p73. However, it is not clear if the specific binding of p28 to these p53 family members could directly increase their expression by inhibiting the proteasome-mediated degradation driven by the ubiquitin ligase COP1, as described for p53 [89]. Accordingly, it is worth to investigate the possible interference of p28 with ubiquitin ligases, controlling the level of p63 and p73 and in particular with COP1, which is the target of p28 activity in protecting p53 from proteasome-mediated degradation and whose role in p63 and p73 regulation is still unknown. 

## 4. Conclusions and Perspectives

**Figure 8:**
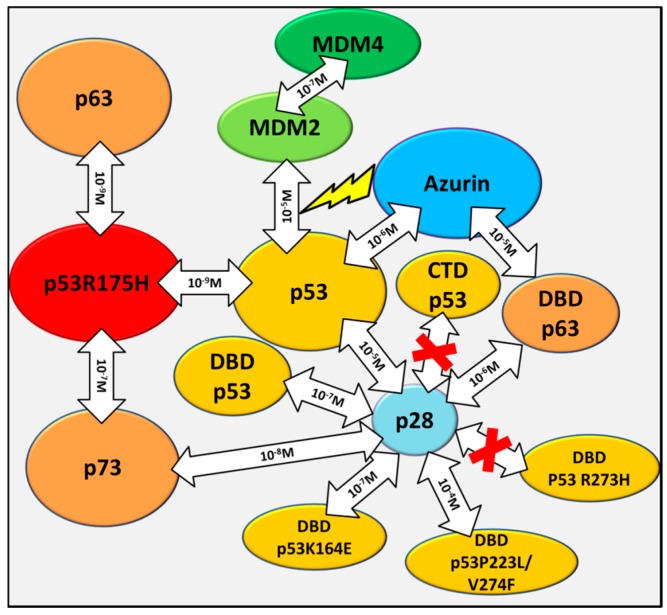
Overall schematic representation of the protein-protein interactions within the p53 network here reviewed; with *K_D_* values being indicated inside the arrows. The red cross indicates no interaction. The “bolt like” arrow indicates the negative allosteric effect exerted by Azurin on the p53-MDM2 interaction.

Applying appropriate immobilization strategies, different analyte injection sequences and suitable models for data analysis, SPR has proven to be a versatile biosensing technique, which allows to determine, in real time and without labelling, reliable and accurate equilibrium kinetic parameters of protein-protein interactions of biomedical interest, including ternary complexes. The SPR results may be supported by the complementary nanotechnological AFS technique, which is also able to provide information on the binding kinetic parameters at the single molecule level. The overview here presented (Figure 8) on the interaction binding kinetics involving p53, its main down-regulators, p53 family members, p63 and p73, some oncogenic mutants and two anticancer drugs, may both contribute to a deeper understanding of the p53 network features and provide useful insights for anticancer drugs strategies. 

SPR sensing could be in perspective exploited to study both the interaction of p53 family members with other inhibitors and the antagonizing effect of novel, or optimized, drugs, which target specific interactions within the p53 network, aimed at restoring its oncosuppressive function. 

## Figures and Tables

**Table 1:** Kinetic parameters of the protein-protein interactions within the p53 network revised in this work.

Protein-Protein Interaction	k_on_ (M^−1^ s^−1^)	k_off_ (s^−1^)	K_D_ (M)	τ (s)	Ref.
MDM2/p53	(0.8 ± 0.3) × 10^6^	(2.1 ± 0.2)	(0.4 ± 0.1) × 10^−6^	(0.5 ± 0.1)	[22]
MDM2/MDM4	(4.3 ± 0.3) × 10^3^	(1.7 ± 0.7) × 10^−3^	(3.9 ± 0.1) × 10^−7^	(6 ± 2) × 10^2^	[23], unp
p53R175H/p53	(1.28 ± 0.04) × 10^4^	(4.6 ± 0.4) × 10^−5^	(3.6 ± 0.3) × 10^−9^	(2.17 ± 0.02) × 10^4^	[24]
p53R175H/p63	(1.52 ± 0.04) × 10^4^	(5.3 ± 0.4) × 10^−5^	(3.5 ± 0.3) × 10^−9^	(1.89 ± 0.01) × 10^4^	[24]
p53R175H/p73	(6.4 ± 0.5) × 10^3^	(3.1 ± 1.8) × 10^−3^	(4.9 ± 0.6) × 10^−7^	(3.2 ± 1.9) × 10^2^	[25]
Az/p53	(8.2 ± 0.5) × 10^4^	(9.4 ± 0.7) × 10^−2^	(1.2 ± 0.1) × 10^−6^	(1.0 ± 0.1) × 10	[22]
Az/(p53-MDM2)	(6.8 ± 0.5) × 10^4^	(9.0 ± 0.3) × 10^−2^	(1.3 ± 0.1) × 10^−6^	(1.0 ± 0.1) × 10	[22]
MDM2/(p53-Az)	(5.0 ± 0.3) × 10^5^	(0.6 ± 0.2)	(1.2 ± 0.4) × 10^−6^	(1.60 ± 0.02)	[22]
p28/p53	(2.4 ± 0.4) × 10^2^	(3.0 ± 0.1) × 10^−3^	(1.2 ± 0.1) × 10^−5^	(3.3 ± 0.3) × 10^2^	unp.
p28/CTDp53	-	-	N.I.	-	unp.
p28/DBDp53	(2.4 ± 0.5) × 10^2^	(2.0 ± 0.1) × 10^−5^	(8.8 ± 1.9) × 10^−8^	(5.0 ± 0.3) × 10^4^	[37], unp.
p28/DBDp53-K164E	-	-	7.3 × 10^−8^	-	[37]
p28/DBDp53-R273H	-	-	N.I.	-	[37]
p28/DBDp53-P223L/V274F	-	-	2.4 × 10^−4^	-	[37]
Az/DBDp63	(4.6 ± 0.5) × 10^2^	(4.2 ± 0.3) × 10^−4^	(8.2 ± 0.3) × 10^−6^	(2.4 ± 0.1) × 10^3^	unp.
p28/DBDp63	(9.2 ± 0.7)	(3.7 ± 0.3) × 10^−5^	(4.2 ± 0.4) × 10^−6^	(2.7 ± 0.2) × 10^4^	unp.
p28/p73	(9.3 ± 0.5) × 10^4^	(1.4 ± 0.1) × 10^−3^	(1.5 ± 0.4) × 10^−8^	(7.1 ± 0.5) × 10^2^	unp.

**Abbreviations:** Azurin (Az), DNA binding domain (DBD), association rate constant (*k_on_*), dissociation rate constant (*k_off_*), equilibrium dissociation constant (*K_D_*), lifetime (τ), no interaction (N.I.); unpublished (unp.), not determined (-).

## References

[1] Nguyen H.H., Park J., Kang S., Kim M. (2015). Surface Plasmon Resonance: A Versatile Technique for Biosensor Applications. Sensors.

[2] Homola J., Yee S.S., Gauglitz G. (1999). Surface plasmon resonance sensors: Review. Sens. Actuators B Chem..

[3] Cooper M.A. (2003). Label-free screening of bio-molecular interactions. Anal. Bioanal. Chem..

[4] Patching S.G. (2014). Surface plasmon resonance spectroscopy for characterisation of membrane protein—Ligand interactions and its potential for drug discovery. Biochim. Biophys. Acta.

[5] Mariani S., Minunni M. (2014). Surface plasmon resonance applications in clinical analysis. Anal. Bioanal. Chem..

[6] Ro H.-S., Koh B.H., Jung S.O., Park H.K., Shin Y.-B., Kim M.-G., Chung B.H. (2006). Surface plasmon resonance imaging protein arrays for analysis of triple protein interactions of HPV, E6, E6AP, and p53. Proteomics.

[7] Aykul S., Martinez-Hackert E. (2016). Determination of half-maximal inhibitory concentration using biosensor-based protein interaction analysis. Anal. Biochem..

[8] Lane D., Levine A. (2010). p53 Research: The past thirty years and the next thirty years. Cold Spring Harb. Perspect. Biol..

[9] Bothner B., Lewis W.S., DiGiammarino E.L., Weber J.D., Bothner S.J., Kriwacki R.W. (2001). Defining the molecular basis of Arf and Hdm2 interactions. J. Mol. Biol..

[10] Burch L.R., Midgley C.A., Currie R.A., Lane D.P., Hupp T.R. (2000). Mdm2 binding to a conformationally sensitive domain on p53 can be modulated by RNA. FEBS Lett..

[11] Chen C., Gorlatova N., Kelman Z., Herzberg O. (2011). Structures of p63 DNA binding domain in complexes with half-site and with spacer-containing full response elements. Proc. Natl. Acad. Sci. USA.

[12] Chen X., Gohain N., Zhan C., Lu W.-Y., Pazgier M., Lu W. (2016). Structural basis of how stress-induced MDMX phosphorylation activates p53. Oncogene.

[13] Kashuba E., Yurchenko M., Yenamandra S.P., Snopok B., Szekely L., Bercovich B., Ciechanover A., Klein G. (2011). Epstein-Barr virus-encoded EBNA-5 forms trimolecular protein complexes with MDM2 and p53 and inhibits the transactivating function of p53. Int. J. Cancer.

[14] Knappskog S., Lønning P.E. (2011). Effects of the MDM2 promoter SNP285 and SNP309 on Sp1 transcription factor binding and cancer risk. Transcription.

[15] Lambert B., Buckle M. (2006). Characterisation of the interface between nucleophosmin (NPM) and p53: Potential role in p53 stabilisation. FEBS Lett..

[16] Lyakhovich A., Shekhar M.P.V. (2003). Supramolecular Complex Formation between Rad6 and Proteins of the p53 Pathway during DNA Damage-Induced Response. Mol. Cell. Biol..

[17] Qin D., Lee H., Yuan C., Ju Y., Tsai M.-D. (2003). Identification of potential binding sites for the FHA domain of human Chk2 by in vitro binding studies. Biochem. Biophys. Res. Commun..

[18] Savchenko A., Yurchenko M., Snopok B., Kashuba E. (2009). Study on the spatial architecture of p53, MDM2, and p14ARF containing complexes. Mol. Biotechnol..

[19] Tomita Y., Marchenko N., Erster S., Nemajerova A., Dehner A., Klein C., Pan H., Kessler H., Pancoska P., Moll U.M. (2006). WT p53, but not tumor-derived mutants, bind to Bcl2 via the DNA binding domain and induce mitochondrial permeabilization. J. Biol. Chem..

[20] Van Dieck J., Lum J.K., Teufel D.P., Fersht A.R. (2010). S100 proteins interact with the N-terminal domain of MDM2. FEBS Lett..

[21] Xia N., Liu L., Yi X., Wang J. (2009). Studies of interaction of tumor suppressor p53 with apo-MT using surface plasmon resonance. Anal. Bioanal. Chem..

[22] Domenici F., Frasconi M., Mazzei F., D’Orazi G., Bizzarri A.R., Cannistraro S. (2011). Azurin modulates the association of Mdm2 with p53: SPR evidence from interaction of the full-length proteins. J. Mol. Recognit..

[23] Moscetti I., Teveroni E., Moretti F., Bizzarri A.R., Cannistraro S. (2016). MDM2–MDM4 molecular interaction investigated by atomic force spectroscopy and surface plasmon resonance. Int. J. Nanomed..

[24] Moscetti I., Bizzarri A.R., Cannistraro S. (2017). Binding kinetics of mutant p53R175H with wild type p53 and p63: A Surface Plasmon Resonance and Atomic Force Spectroscopy study. Biophys. Chem..

[25] Santini S., Di Agostino S., Coppari E., Bizzarri A.R., Blandino G., Cannistraro S. (2014). Interaction of mutant p53 with p73: A Surface Plasmon Resonance and Atomic Force Spectroscopy study. Biochim. Biophys. Acta.

[26] Azmi A.S., Philip P.A., Wang Z., Banerjee S., Zafar S.F., Goustin A.-S., Almhanna K., Yang D., Wang S., Sarkar F.H., Mohammad R.M. (2010). Reactivation of p53 by Novel MDM2 Inhibitors: Implications for Pancreatic Cancer Therapy. Curr. Cancer Drug Targets.

[27] Bechill J., Zhong R., Zhang C., Solomaha E., Spiotto M.T. (2016). A High-Throughput Cell-Based Screen Identified a 2-[(E)-2-Phenylvinyl]-8-Quinolinol Core Structure That Activates p53. PLoS ONE.

[28] Danelius E., Pettersson M., Bred M., Min J., Waddell M.B., Guy R.K., Grøtli M., Erdelyi M. (2016). Flexibility is important for inhibition of the MDM2/p53 protein-protein interaction by cyclic β-hairpins. Org. Biomol. Chem..

[29] Fry D.C., Wartchow C., Graves B., Janson C., Lukacs C., Kammlott U., Belunis C., Palme S., Klein C., Vu B. (2013). Deconstruction of a Nutlin: Dissecting the Binding Determinants of a Potent Protein–Protein Interaction Inhibitor. ACS Med. Chem. Lett..

[30] Huang A., Zhou L., Zhang D., Yao J., Zhang Y. (2016). Molecular design and validation of halogen bonding orthogonal to hydrogen bonding in breast cancer MDM2-peptide complex. J. Mol. Graph. Model..

[31] Kikuchi J., Furukawa Y., Hayashi N. (2003). Identification of novel p53-binding proteins by biomolecular interaction analysis combined with tandem mass spectrometry. Mol. Biotechnol..

[32] Lv C., Hong Y., Miao L., Li C., Xu G., Wei S., Wang B., Huang C., Jiao B. (2013). Wentilactone A as a novel potential antitumor agent induces apoptosis and G2/M arrest of human lung carcinoma cells, and is mediated by HRas-GTP accumulation to excessively activate the Ras/Raf/ERK/p53-p21 pathway. Cell Death Dis..

[33] Okuda Y., Nakamura H.K., Kuwata K. (2009). Novel anti-cancer compounds: Structure-based discovery of chemical chaperons for p53. Oncol. Rep..

[34] Pazgier M., Liu M., Zou G., Yuan W., Li C., Li C., Li J., Monbo J., Zella D., Tarasov S.G., Lu W. (2009). Structural basis for high-affinity peptide inhibition of p53 interactions with MDM2 and MDMX. Proc. Natl. Acad. Sci. USA.

[35] Roxburgh P., Hock A.K., Dickens M.P., Mezna M., Fischer P.M., Vousden K.H. (2012). Small molecules that bind the Mdm2 RING stabilize and activate p53. Carcinogenesis.

[36] Smith J.M., Frost J.R., Fasan R. (2014). Designer macrocyclic organo-peptide hybrids inhibit the interaction between p53 and HDM2/X by accommodating a functional α-helix. Chem. Commun. Camb. Engl..

[37] Signorelli S., Santini S., Yamada T., Bizzarri A.R., Beattie C.W., Cannistraro S. (2017). Binding of Amphipathic Cell Penetrating Peptide p28 to Wild Type and Mutated p53 as studied by Raman, Atomic Force and Surface Plasmon Resonance spectroscopies. Biochim. Biophys. Acta.

[38] Bizzarri A.R., Cannistraro S. (2010). The application of atomic force spectroscopy to the study of biological complexes undergoing a biorecognition process. Chem. Soc. Rev..

[39] Bizzarri A.R., Cannistraro S. (2009). Atomic Force Spectroscopy in Biological Complex Formation: Strategies and Perspectives. J. Phys. Chem. B.

[40] Hinterdorfer P., Dufrêne Y.F. (2006). Detection and localization of single molecular recognition events using atomic force microscopy. Nat. Methods.

[41] Van Der Merwe P.A. (2001). Surface Plasmon Resonance.

[42] Schasfoort R.B.M. (2017). Handbook of Surface Plasmon Resonance.

[43] Karlsson R., Katsamba P.S., Nordin H., Pol E., Myszka D.G. (2006). Analyzing a kinetic titration series using affinity biosensors. Anal. Biochem..

[44] Björquist P., Boström S. (1997). Determination of the kinetic constants of tissue factor/factor VII/factor VIIA and antithrombin/heparin using surface plasmon resonance. Thromb. Res..

[45] O’Shannessy D.J., Brigham-Burke M., Soneson K.K., Hensley P., Brooks I. (1993). Determination of rate and equilibrium binding constants for macromolecular interactions using surface plasmon resonance: Use of nonlinear least squares analysis methods. Anal. Biochem..

[46] Glaser R.W. (1993). Antigen-antibody binding and mass transport by convection and diffusion to a surface: A two-dimensional computer model of binding and dissociation kinetics. Anal. Biochem..

[47] Morton T.A., Myszka D.G., Chaiken I.M. (1995). Interpreting complex binding kinetics from optical biosensors: A comparison of analysis by linearization, the integrated rate equation, and numerical integration. Anal. Biochem..

[48] Myszka D.G., Morton T.A. (1998). CLAMP: A biosensor kinetic data analysis program. Trends Biochem. Sci..

[49] Shangary S., Wang S. (2008). Targeting the MDM2-p53 Interaction for Cancer Therapy. Clin. Cancer Res. Off. J. Am. Assoc. Cancer Res..

[50] Funari G., Domenici F., Nardinocchi L., Puca R., D’Orazi G., Bizzarri A.R., Cannistraro S. (2010). Interaction of p53 with Mdm2 and azurin as studied by atomic force spectroscopy. J. Mol. Recognit..

[51] Chen R., Zhou J., Qin L., Chen Y., Huang Y., Liu H., Su Z. (2017). A Fusion Protein of the p53 Transaction Domain and the p53-Binding Domain of the Oncoprotein MdmX as an Efficient System for High-Throughput Screening of MdmX Inhibitors. Biochemistry (Moscow).

[52] Dawson R., Müller L., Dehner A., Klein C., Kessler H., Buchner J. (2003). The N-terminal domain of p53 is natively unfolded. J. Mol. Biol..

[53] Wang X., Jiang X. (2012). Mdm2 and MdmX partner to regulate p53. FEBS Lett..

[54] Huang L., Yan Z., Liao X., Li Y., Yang J., Wang Z.-G., Zuo Y., Kawai H., Shadfan M., Ganapathy S., Yuan Z.-M. (2011). The p53 inhibitors MDM2/MDMX complex is required for control of p53 activity in vivo. Proc. Natl. Acad. Sci. USA.

[55] Pant V., Xiong S., Iwakuma T., Quintás-Cardama A., Lozano G. (2011). Heterodimerization of Mdm2 and Mdm4 is critical for regulating p53 activity during embryogenesis but dispensable for p53 and Mdm2 stability. Proc. Natl. Acad. Sci. USA.

[56] Wade M., Li Y.-C., Wahl G.M. (2013). MDM2, MDMX and p53 in oncogenesis and cancer therapy. Nat. Rev. Cancer.

[57] Sharp D.A., Kratowicz S.A., Sank M.J., George D.L. (1999). Stabilization of the MDM2 oncoprotein by interaction with the structurally related MDMX protein. J. Biol. Chem..

[58] Tanimura S., Ohtsuka S., Mitsui K., Shirouzu K., Yoshimura A., Ohtsubo M. (1999). MDM2 interacts with MDMX through their RING finger domains. FEBS Lett..

[59] Collavin L., Lunardi A., Del Sal G. (2010). p53-family proteins and their regulators: Hubs and spokes in tumor suppression. Cell Death Differ..

[60] Deyoung M.P., Ellisen L.W. (2007). p63 and p73 in human cancer: Defining the network. Oncogene.

[61] Petitjean A., Mathe E., Kato S., Ishioka C., Tavtigian S.V., Hainaut P., Olivier M. (2007). Impact of mutant p53 functional properties on TP53 mutation patterns and tumor phenotype: Lessons from recent developments in the IARC TP53 database. Hum. Mutat..

[62] Di Como C.J., Gaiddon C., Prives C. (1999). p73 function is inhibited by tumor-derived p53 mutants in mammalian cells. Mol. Cell. Biol..

[63] Gaiddon C., Lokshin M., Ahn J., Zhang T., Prives C. (2001). A subset of tumor-derived mutant forms of p53 down-regulate p63 and p73 through a direct interaction with the p53 core domain. Mol. Cell. Biol..

[64] Strano S., Munarriz E., Rossi M., Cristofanelli B., Shaul Y., Castagnoli L., Levine A.J., Sacchi A., Cesareni G., Oren M. (2000). Physical and functional interaction between p53 mutants and different isoforms of p73. J. Biol. Chem..

[65] Strano S., Fontemaggi G., Costanzo A., Rizzo M.G., Monti O., Baccarini A., Del Sal G., Levrero M., Sacchi A., Oren M. (2002). Physical interaction with human tumor-derived p53 mutants inhibits p63 activities. J. Biol. Chem..

[66] Xu J., Reumers J., Couceiro J.R., De Smet F., Gallardo R., Rudyak S., Cornelis A., Rozenski J., Zwolinska A., Marine J.-C. (2011). Gain of function of mutant p53 by coaggregation with multiple tumor suppressors. Nat. Chem. Biol..

[67] Service R.F. (2016). Rescuing the guardian of the genome. Science.

[68] Kehrloesser S., Osterburg C., Tuppi M., Schäfer B., Vousden K.H., Dötsch V. (2016). Intrinsic aggregation propensity of the p63 and p73 TI domains correlates with p53R175H interaction and suggests further significance of aggregation events in the p53 family. Cell Death Differ..

[69] Wang G., Fersht A.R. (2015). Propagation of aggregated p53: Cross-reaction and coaggregation vs. seeding. Proc. Natl. Acad. Sci. USA.

[70] Billant O., Léon A., Le Guellec S., Friocourt G., Blondel M., Voisset C. (2016). The dominant-negative interplay between p53, p63 and p73: A family affair. Oncotarget.

[71] Rajagopalan S., Huang F., Fersht A.R. (2011). Single-Molecule characterization of oligomerization kinetics and equilibria of the tumor suppressor p53. Nucleic Acids Res..

[72] Gaglia G., Guan Y., Shah J.V., Lahav G. (2013). Activation and control of p53 tetramerization in individual living cells. Proc. Natl. Acad. Sci. USA.

[73] Punj V., Bhattacharyya S., Saint-Dic D., Vasu C., Cunningham E.A., Graves J., Yamada T., Constantinou A.I., Christov K., White B. (2004). Bacterial cupredoxin azurin as an inducer of apoptosis and regression in human breast cancer. Oncogene.

[74] Yamada T., Goto M., Punj V., Zaborina O., Chen M.L., Kimbara K., Majumdar D., Cunningham E., Das Gupta T.K., Chakrabarty A.M. (2002). Bacterial redox protein azurin, tumor suppressor protein p53, and regression of cancer. Proc. Natl. Acad. Sci. USA.

[75] Taranta M., Bizzarri A.R., Cannistraro S. (2008). Probing the interaction between p53 and the bacterial protein azurin by single molecule force spectroscopy. J. Mol. Recognit..

[76] Gabellieri E., Bucciantini M., Stefani M., Cioni P. (2011). Does azurin bind to the transactivation domain of p53? A Trp phosphorescence study. Biophys. Chem..

[77] Apiyo D., Wittung-Stafshede P. (2005). Unique complex between bacterial azurin and tumor-suppressor protein p53. Biochem. Biophys. Res. Commun..

[78] Kussie P.H., Gorina S., Marechal V., Elenbaas B., Moreau J., Levine A.J., Pavletich N.P. (1996). Structure of the MDM2 Oncoprotein Bound to the P53 Tumor Suppressor Transactivation Domain. Science.

[79] Chi S.-W., Lee S.-H., Kim D.-H., Ahn M.-J., Kim J.-S., Woo J.-Y., Torizawa T., Kainosho M., Han K.-H. (2005). Structural details on mdm2-p53 interaction. J. Biol. Chem..

[80] De Grandis V., Bizzarri A.R., Cannistraro S. (2007). Docking study and free energy simulation of the complex between p53 DNA-binding domain and azurin. J. Mol. Recognit..

[81] Bizzarri A.R., Di Agostino S., Andolfi L., Cannistraro S. (2009). A combined atomic force microscopy imaging and docking study to investigate the complex between p53 DNA binding domain and Azurin. J. Mol. Recognit..

[82] Yamada T., Mehta R.R., Lekmine F., Christov K., King M.L., Majumdar D., Shilkaitis A., Green A., Bratescu L., Beattie C.W. (2009). A peptide fragment of azurin induces a p53-mediated cell cycle arrest in human breast cancer cells. Mol. Cancer Ther..

[83] Lulla R.R., Goldman S., Yamada T., Beattie C.W., Bressler L., Pacini M., Pollack I.F., Fisher P.G., Packer R.J., Dunkel I.J. (2016). Phase I trial of p28 (NSC745104), a non-HDM2-mediated peptide inhibitor of p53 ubiquitination in pediatric patients with recurrent or progressive central nervous system tumors: A Pediatric Brain Tumor Consortium Study. Neuro-Oncology.

[84] Warso M.A., Richards J.M., Mehta D., Christov K., Schaeffer C., Rae Bressler L., Yamada T., Majumdar D., Kennedy S.A., Beattie C.W. (2013). A first-in-class, first-in-human, phase I trial of p28, a non-HDM2-mediated peptide inhibitor of p53 ubiquitination in patients with advanced solid tumours. Br. J. Cancer.

[85] Yamada T., Christov K., Shilkaitis A., Bratescu L., Green A., Santini S., Bizzarri A.R., Cannistraro S., Gupta T.K.D., Beattie C.W. (2013). p28, a first in class peptide inhibitor of cop1 binding to p53. Br. J. Cancer.

[86] Yamada T., Das Gupta T.K., Beattie C.W. (2013). p28, an anionic cell-penetrating peptide, increases the activity of wild type and mutated p53 without altering its conformation. Mol. Pharm..

[87] Bizzarri A.R., Santini S., Coppari E., Bucciantini M., Di Agostino S., Yamada T., Beattie C.W., Cannistraro S. (2011). Interaction of an anticancer peptide fragment of azurin with p53 and its isolated domains studied by atomic force spectroscopy. Int. J. Nanomed..

[88] Santini S., Bizzarri A.R., Cannistraro S. (2011). Modelling the interaction between the p53 DNA-binding domain and the p28 peptide fragment of Azurin. J. Mol. Recognit..

[89] Coppari E., Yamada T., Bizzarri A.R., Beattie C.W., Cannistraro S. (2014). A nanotechnological, molecular-modeling, and immunological approach to study the interaction of the anti-tumorigenic peptide p28 with the p53 family of proteins. Int. J. Nanomed..

